# Reproductive life disorders in Italian celiac women. A case-control study

**DOI:** 10.1186/1471-230X-10-89

**Published:** 2010-08-06

**Authors:** Domenico Martinelli, Francesca Fortunato, Silvio Tafuri, Cinzia A Germinario, Rosa Prato

**Affiliations:** 1 Section of Hygiene, Department of Medical and Occupational Science, University of Foggia, Viale Pinto, 71000 Foggia, Italy; 2 Section of Hygiene, Department of Biomedical Sciences and Human Oncology, University of Bari, Piazza Giulio Cesare, 11, 70124 Bari, Italy

## Abstract

**Background:**

The aim of this study is to explore the association between celiac disease and menstrual cycle, gestation and puerperal disorders.

**Methods:**

The association between celiac disease and menstrual cycle, gestation and puerperal disorders in a sample of 62 childbearing age women (15-49 age) was assessed within an age and town of residence matched case-control study conducted in 2008. Main outcome measures were the presence of one or more disorders in menstrual cycle and the presence of one or more complication during pregnancy.

**Results:**

62 celiac women (median age: 31.5, range: 17-49) and 186 healthy control (median age: 32.5, range: 15-49) were interviewed. A higher percentage of menstrual cycle disorders has been observed in celiac women. 19.4% frequency of amenorrhea was reported among celiac women versus 2.2% among healthy controls (OR = 33, 95% CI = 7.17-151.8;, p = 0.000). An association has been observed between celiac disease and oligomenorrhea, hypomenorrhea, dysmenorrhea and metrorrhagia (p < 0.05). The likelihood of having at least one complication during pregnancy has been estimated to be at least four times higher in celiac women than in healthy women (OR = 4.1, 95% CI = 2-8.6, p = 0.000). A significant correlation has emerged for celiac disease and threatened abortion, gestational hypertension, placenta abruption, severe anaemia, uterine hyperkinesia, intrauterine growth restriction (p < 0.001). A shorter gestation has on average been observed in celiac women together with a lower birth weight of celiac women babies (p < 0.001).

**Conclusions:**

The occurrence of a significant correlation between celiac disease and reproductive disorders could suggest to consider celiac disease diagnostic procedures (serological screening) in women affected by these disorders.

## Background

Celiac Disease (CD), also known as celiac sprue or gluten enteropathy, is a permanent intolerance to gluten, a protein complex contained in wheat, barley and rye, characterized by a wide clinical variability. In genetically predisposed subjects, exposure to gluten results in a mucosal damage which, progressing through different stages of severity, causes small-intestinal mucosal atrophy. A diet completely free of the above cereals results in the total resolution of the clinical picture as well as in complete healing of jejunal mucosa histological lesions [1].

Celiac disease occurs in adults and children with an incidence approaching 1% of population in Western countries [2]. Recent studies have shown that celiac disease could be more frequent than previously expected in Africa, South America, and Asia [3-6]. In a study published in 2001, Volta *et al. *reported a CD prevalence of about one case in 170 inhabitants in Italy with a female predilection (women to men ratio = 2.3:1) [7,8]. In a previous multi-centric study carried out by Catassi *et al. *on more than 17,000 students (age range: 6-15) the prevalence of celiac disease was observed to be of one case in 184 students with an elevated incidence of atypical forms [9].

Several studies have shown that celiac disease can impair women's reproductive life eliciting delayed puberty, infertility, amenorrhea and early menopause. Some clinical and epidemiological studies have demonstrated that women with celiac disease are at a higher risk of miscarriage, low birth weight of the newborn [10-13].

In particular, in some studies the age at menarche of celiac women has been found to be older compared to that of healthy controls, whereas the average age at menopause of celiac women has been observed to be younger than that of healthy women; in the same studies an increased frequency of cases of secondary amenorrhea has also been registered among celiac women [14].

A study conducted in 1996 by Ciacci *et al. *at the University of Naples highlighted a remarkable increase in the incidence of miscarriages in pregnant celiac women with an almost 9-fold increased risk compared to non celiac women [15]. This finding confirmed the results of one of the first studies on the effects of CD on pregnancy which was conducted in 1975 by Ogborn A.D.R. where non treated celiac women were observed to exhibit a 7-fold increased risk of miscarriage compared to that of celiac women on a Gluten Free Diet (GFD) [16]. In a 1996 study carried out at the Hospital Municipal de Gastroenterelogia (Salvador University, Buenos Aires - Argentina) a Relative Risk of 5.8 for low birth weight was reported for the children of celiac women [17]. On the contrary, in 1990 Molteni *et al. *did not observe any significant differences in the incidence of low-birth-weight newborns before (16%) and after a GFD [14]. A group of 12 pregnant women with CD enrolled in a study conducted by Martinelli *et al. *in 2000 gave birth to three severely premature newborns (25%) and five underweight newborns (41%). In addition, three cases of breech presentation at delivery (25%) and one case of pre-eclampsia were observed in the same group [18]. In a recent paper, Khashan AS *et al. *reported that women with untreated CD delivered babies with a higher risk of small for gestational age (OR = 1.31, 95% CI: 1.06, 1.63) and very small for gestational age infants (OR = 1.54, 95% CI: 1.17, 2.03) and preterm birth (OR = 1.33, 95% CI: 1.02, 1.72) [19].

The aim of the present study is to explore the association between celiac disease and menstrual cycle, gestation and puerperal disorders in childbearing-age women resident in Puglia (Southern Italy, 4 million of inhabitants).

## Methods

The association between celiac disease and menstrual cycle, gestation and puerperal disorders in the women of childbearing age of Apulia was assessed within a matched case-control study conducted between April and December 2008. The Protocol of the Study in question was submitted for approval to the Technical-Scientific Committee of the Regional Epidemiologic Observatory. Approval was given and permission was granted to use the results of the tests anonymously for scientific aims on February 20, 2008. The research was carried out in compliance with the Helsinki Declaration.

The inclusion criteria for the sample of celiac women were as follows: "woman 15-49 years old residing in Apulia at the time of the study with a diagnosis of celiac disease by a medical specialist relying on one or more specific tests (serum levels of anti-gliadin and/or anti-endomysial, and/or anti-transglutamminase antibodies and/or small intestine biopsy)" [20,21].

The women enrolled were selected among those participating in a meeting of the Apulian Section of the Italian Association of Celiac Disease (AIC) which took place in Apulia in April 2008. AIC meets patients with different degree of CD to promote the assistance of them and their families and scientific research. After subscribing to an informed consent, all the women of the sample in question were administered a standardized questionnaire.

Main investigated outcomes were history of menstrual cycle disorders and history of pregnancy problems (Additional file 1).

The group of controls was randomly selected from the Register Citizens with eligibility for Regional Health Services: three age-matched (15-29, 30-39 and 40-49) and town of residence-matched healthy controls (women with no diagnosis of celiac disease) were selected for each celiac woman. Three potential replacements were selected for each control. All controls were reached on the phone: after accepting to go on with the interview, they were all administered the same standardized questionnaire used for cases (without the questions about celiac disease).

After each interview the personal data of the respondents were cancelled, in full compliance with the privacy law in force. The data derived from the questionnaires were then gathered in an electronic database (Access format).

To evaluate potential associations across the variables explored double-entry contingency tables (2 × 2) were defined and the chi-square (χ^2^) value determined by considering as significant p values <0.05. Fisher's exact test was performed when an expected variable value was less than 5. A matched analysis was used to calculate the Odds Ratio (OR) and related coverage probabilities for 95% Confidence Intervals (CI). The assessment of significant differences across the means of continuous variables (age at menarche, gestation week, birth weight, breastfeeding duration) relied on the t-test for independent samples considering as significant those values with p < 0.05. Data were processed by Epi Info 3.0 software.

## Results

All the participants at the convention in the 15-49 age group, accepted to be enrolled in the study. A total number of 62 interviews of celiac women (average age: 33.2 ± 9.7, 95% CI: 30.7-35.7) were collected. The response rate was 100%. 35.5% of the women resided in the province of Bari, 30.6% in the province of Foggia, 14.5% in the province of Lecce, 12.9% in the province of Taranto, 4.8% in the province of BAT, 1.6% in the province of Brindisi (Table 1). Three age-matched and town of residence-matched controls were interviewed for each celiac woman: a total number of 186 interviews were collected with a replacement rate of about 15%. The commonest reason for replacement was refusal to join the study. The average age of controls (32.8 ± 9.9 years, 95% CI: 31.4-34.3) and their distribution *per *province turned out to overlap those of celiac women (Table 1).

**Table 1:** Sample description, by age classes and provinces of residence.

Age classes		Cases		Controls	
			
		n°	*%*	n°	*%*
	15-29	25	*40.3%*	75	*40.3%*
	30-39	16	*25.8%*	48	*25.8%*
	40-49	21	*33.9%*	63	*33.9%*
	
	Total	62	-	186	-

Provinces of residence		n°	*%*	n°	*%*

	Bari	22	*35.5%*	66	*35.5%*
	BAT	3	*4.8%*	9	*4.8%*
	Brindisi	1	*1.6%*	3	*1.6%*
	Foggia	19	*30.6%*	57	*30.6%*
	Lecce	9	*14.5%*	27	*14.5%*
	Taranto	8	*12.9%*	24	*12.9%*
	
	Total	62	-	186	-

In 100% of cases (n = 62) the diagnosis of celiac disease had been made by a medical specialist: the serum levels of anti-gliadin antibodies (AGA) had been measured in 58.1% of the cases of celiac disease (n = 36), anti-endomysial antibodies (AEA) had been assessed in 56.5% of celiac women (n = 35), and anti-transglutamminase antibodies (anti-tTG) had been dosed in 41.9% (n = 26) of them. 41.9% (n = 26) of the celiac women interviewed had undergone small intestine biopsy. In one case HLA genomic typing for DQ2/DQ8 had been performed.

The average age at the diagnosis of celiac disease of the women interviewed turned out to be 24.2 ± 13.57 (95% CI: 20.7-27.6; range: 1 year - 47 years).

In 69.3% (n = 43) of cases the onset symptom had been "bloated stomach", in 61.3% (n = 38) it had been "anaemia", in 51.6% (n = 32) "weight loss", in 40.3% (n = 25) "diarrhea", in 17.7% (n = 11) "vomiting"; 40.3% (n = 25) of cases had presented with "other symptoms/signs" including "menstrual cycle disorders" (4.8% of cases). 8.1% (n = 5) of celiac women reported that more than four symptoms were present at the onset of their disease, whereas in 45.2% (n = 30) of them onset symptoms/signs had been three or four and one or two in 46.7% (n = 29).

At the time of the interview 98.4% (n = 61) of the celiac women had been on a gluten free diet (GFD) for on average 7.9 ± 7.8 years (95% CI: 6-10 years).

The average age at menarche of celiac women was 12.7 ± 1.4 years (95% CI: 12.3-13.0), overlapping that of controls (12.4 ± 1.4 years; 95% CI: 12.2-12.6, p = 0.1838).

67.7% (n = 42) of celiac women and 64.5% (n = 120) of controls reported a past history of at least one menstrual cycle disorder (p = 0.6456). Table 2 reports menstrual cycle disorders prevalence.

**Table 2:** Menstrual cycle disorders prevalence (OR and 95% CI).

	Cases		Controls		OR *(95% CI)*	**χ**^**2**^	p
					
Menstrual cycle disorders	n°	*%*	n°	*%*			
No	20	*32.3*	66	*35.5*	1.2 (*0.6-2.3*)	0.09	0.6456
Yes	42	*67.7*	120	*64.5*			
			
Total	62	-	186	-			
	
Amenorrhea							

No	50	*80.6*	182	*97.8*	33 (*7.2-151.8*)	*	0.0000
Yes	12	*19.4*	4	*2.2*			
			
Total	62	-	186	-			
	
Oligomenorrhea							

No	48	*77.4*	164	*88.2*	2.7 (*1.1-6.4*)	3.37	0.0412
Yes	14	*22.6*	22	*11.8*			
			
Total	62	-	186	-			
	
Hypomenorrhea							

No	48	*77.4*	176	*94.6*	9.8 (*3.3-29.4*)	13.23	0.0001
Yes	14	*22.6*	10	*5.4*			
			
Total	62	-	186	-			
	
Polymenorrhea							

No	57	*91.9*	179	*96.2*	3.28 (*0.7-14.7*)	1.12	0.01573
Yes	5	*8.1*	7	*3.8*			
			
Total	62	-	186	-			

Dysmenorrhea							

No	32	*51.6*	126	*67.7*	2.3 (*1.2-4.4*)	4.61	0.0214
Yes	30	*48.4*	60	*32.3*			
			
Total	62	-	186	-			

Metrorrhagia							

No	56	*90.3*	182	*97.8*	9.75 (*1.9-50.6*)	4.80	0.0106
Yes	6	*9.7*	4	*2.2*			
			
Total	62	-	186	-			

Menometrorrhagia							

No	54	*87.1*	148	*79.6*	0.52 (*0.2-1.3*)	2.28	0.1859
Yes	8	*12.9*	38	*20.4*			
			
Total	62	-	186	-			

Pre-menstrual syndrome							

No	32	*51.6*	97	*52.2*	1.02 (*0.5-1.9*)	0.0058	0.9394
Yes	30	*48.4*	89	*47.8*			
			
Total	62	-	186	-			

*Fischer's exact test used to assess p; 95% CI poorly accurate.

**Table 3:** Gestational disorders prevalence (OR and 95% CI).

		Cases		Controls		OR *(95% CI)*	**χ**^**2**^	p
						
At least one gestational disorder		n°	*%*	n°	*%*			
	No	17	*34.7*	123	*68.7*	4.1 (*2-8.6*)	4.1	0.0000
	Yes	32	*65.3*	56	*31.3*			
				
	Total	49	-	179	-			
	
Threatened abortion		n°	*%*	n°	*%*			

	No	30	*61.2*	163	*91.1*	6.4 (*2.8-15*)	26.4	0.0000
	Yes	19	*38.8*	16	*8.9*			
				
	Total	49	-	179	-			
	
Gestational hypertension		n°	*%*	n°	*%*			

	No	44	*89.0*	179	*100.0*	-	*	0.0004
	Yes	5	*10.2*	0	*0.0*			
				
	Total	49	-	179	-			
	
Placenta abruption		n°	*%*	n°	*%*			

	No	40	*81.6*	177	*98.9*	19.9 (*3.8-192.7*)	*	0.0000
	Yes	9	*18.4*	2	*1.1*			
				
	Total	49	-	179	-			
	
Severe anemia		n°	*%*	n°	*%*			

	No	29	*59.2*	175	*97.8*	30.2 (*9-127*)	*	0.0000
	Yes	20	*40.8*	4	*2.2*			
				
	Total	49	-	179	-			
	
Uterine hyperkinesia		n°	*%*	n°	*%*			

	No	44	*89.8*	179	*100.0*	-	*	0.0004
	Yes	5	*10.2*	0	*0.0*			
				
	Total	49	-	179	-			
	
Intrauterine growth restriction		n°	*%*	n°	*%*			

	No	46	*93.8*	179	*100.0*	-	*	0.0095
	Yes	3	*6.2*	0	*0.0*			
				
	Total	49	-	179	-			

*Fischer's exact test used to assess p; 95% CI poorly accurate.

**Figure 1:**
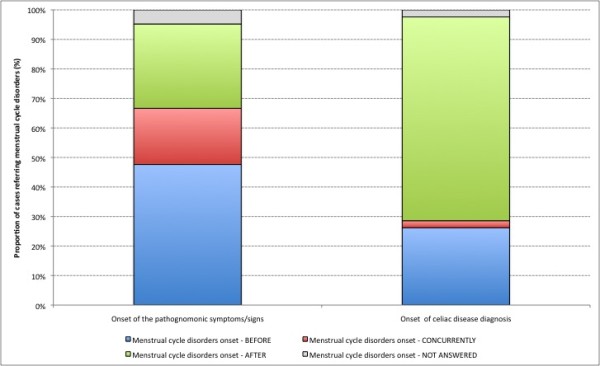
**Proportion of cases referring menstrual cycle disorders, by onset time of menstrual cycle disorders over onset time of the pathognomonic symptoms/signs of celiac disease and onset time of celiac disease diagnosis**.

47.6% (n = 20) of the women with celiac disease who reported they had suffered from menstrual cycle disorders had observed the onset of these latter "before" that of other celiac disease-ascribable symptoms/signs; 28.6% of them had seen the onset of the same menstrual cycle disorders "after" other celiac disease-traceable symptoms/signs; whereas in 19% (n = 8) of celiac women menstrual cycle disorders had appeared "concurrently" with other symptoms/signs (Figure 1). Two celiac women did not specify any time relation between celiac disease-ascribable symptoms/signs and menstrual cycle disorders. 26.2% (n = 11) of celiac women who reported menstrual cycle disorders had received a diagnosis of celiac disease "before" the onset of the same menstrual cycle disorders; whereas 69.1% of them had been diagnosed celiac disease "after" the onset of menstrual cycle disorders, in one case only diagnosis and onset of menstrual cycle disorders had been "concurrent" (Figure 1). One celiac woman did not specify any time relation between the onset of menstrual cycle disorders and celiac disease diagnosis. 73.8% (n = 31) of celiac women were not on a gluten free diet at the onset of the menstrual cycle disorders reported.

At the time of the interview 41.9% (n = 26) of celiac women and 51.1% (n = 95) of controls reported they still suffered from menstrual cycle disorders (p = 0.2140).

50% (n = 31) of celiac women and 50% (n = 93) of controls had had one or more pregnancies. Celiac women reported a total number of 63 pregnancies, 49 (77.8%) of which had been brought to full term, while controls reported a total of 203 pregnancies, 179 (88.2%) of which had been brought to full term. 22.2% (n = 14) of the pregnancies of celiac women had ended in a miscarriage versus 11.8% (n = 24) of the pregnancies of controls (OR = 2.1, 95% CI = 0.9-4.6; χ^2 ^= 4.2, p = 0.0393).

**Figure 2:**
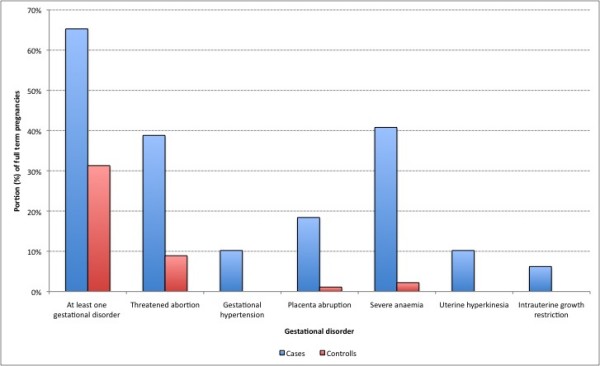
**Proportion of pregnancies, by gestational disorder**.

In 65.3% (n = 32) of the celiac women full term pregnancies at least one gestational disorder was reported versus 31.3% (n = 56) in the full term pregnancies of controls (OR = 4.1, 95% CI = 2-8.6; χ^2 ^= 18.8, p = 0.000). Table 2 and Figure 2 report gestational disorders prevalence and proportion of pregnancies, by gestational disorder respectively.

**Figure 3:**
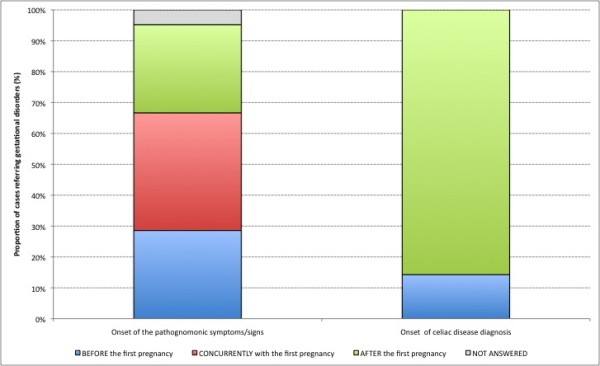
**Proportion of cases referring gestational disorders, by time of onset of celiac disease pathognomonic symptoms/signs and celiac disease diagnosis**.

28.6% (n = 6) of the celiac women who reported problems during pregnancies had exhibited celiac disease-ascribable symptoms/signs before their first pregnancy; 38.1% (n = 8) reported problems "after" their first pregnancy and 28.6% (n = 6) had had their symptoms/signs "concurrently" with their first pregnancy (Figure 3). One of the celiac women did not specify any time relation between celiac disease-ascribable symptoms and her first pregnancy.

14.3% (n = 3) of the celiac women with pregnancy problems had been diagnosed with CD before their first pregnancy, while 85.7% (n = 21) of them had received their diagnosis "after" their first pregnancy (Figure 3). A gluten free diet had been followed during 15 pregnancies (23.8%).

Gestation duration in celiac women was on average 38.5 ± 2.6 weeks (95% CI: 37.7-39.2), versus 40.5 ± 3.6 weeks (95% CI: 39.9-41; t = 3.6, p = 0.000) in healthy controls.

The average birth weight of the children of the celiac women (2956.7 ± 587.3 grams, 95% CI: 2788.0-3125.4) was lower than that of the children of healthy controls (3271.6 ± 637.6 grams, 95% CI: 3177.5-3365.6; t = 3.1, p = 0.000).

The percentage of breastfed babies has been observed to overlap between celiac women (81.6%) and healthy controls (79.3%; OR: 1.2, 95% CI: 0.5-3; χ^2 ^= 0.36, p = 0.5499), whereas the duration of breastfeeding has been found to be shorter (146.4 ± 104.7 days, 95% CI: 112.9-179.9) in celiac women compared to that of healthy women (229.8 ± 177.7 days, 95% CI: 200.4-259.4; t = 2.8, p = 0.008).

## Discussion and conclusions

Several studies have shown an association between celiac disease and women's fertility disorders or pregnancy and puerperal disorders [10-18,22], whereas some other studies have assumed a potential role for celiac disease among the other causes of women's infertility [23]. Instead in a study published in 2005, Tata *et al. *affirmed that, women with celiac disease have fertility similar to that of the general female population, but they have their babies at an older age [24].

The pathogenesis of celiac disease-related reproductive disorders still awaits clarification. Several hypothesis have been proposed to explain reproductive life disorders in women. CD can induce malabsorption and deficiencies of micronutrients such as iron, folic acid and vitamin K, which are essential for organogenesis. Moreover, the deficiencies of specific trace elements could be involve in ovarian dysfunction [25,26]. Finally, in a paper published in 2004, Kotze LM suggested that gluten per se could explain the disturbances and malnutrition would worsen the disease in a consequent vicious cycle [27].

In our study the average age at menarche of celiac women has turned out to overlap that of healthy women; whereas, in line with literature data, a higher percentage of menstrual cycle disorders has been observed in celiac women. In particular, our study confirms the association between celiac disease and amenorrhea [14], with a 19.4% frequency of this disorder among celiac women versus 2.2% among healthy controls. What is more, an association has been observed between celiac disease and oligomenorrhea, hypomenorrhea, dysmenorrhea and metrorrhagia.

Although only a small number (4.8%) of the celiac women enrolled in our study have reported a menstrual cycle disorder as the onset symptom of their disease, almost half of the celiac women with at least one menstrual cycle disorder have reported that this/these latter appeared before the other typical symptoms/signs of celiac disease (Figure 1). Almost 70% of the celiac women with menstrual cycle disorders have reported that their diagnosis of CD had been made after the onset of menstrual alterations. 70% of the celiac women with menstrual cycle disorders have reported that they did not follow a gluten free diet. Although in our study it has not been possible to perform a direct assessment of the protective effect of a GFD on the onset of menstrual cycle disorders, the results obtained seem to substantiate a possible relation between the two, as already shown for pregnancy disorders [16,24-28].

Our study has also showed an association between celiac disease and pregnancy disorders. The number of pregnancies ended in a miscarriage in celiac women has been observed to be almost twice that of healthy women, although the association in question is not statistically significant. In general, a larger number of complications have been observed in celiac women full term pregnancies compared to healthy women. In particular, the likelihood of having at least one complication during pregnancy has been estimated to be at least four times higher in celiac women than in healthy women. Moreover, a significant correlation has emerged for all the disorders under consideration (threatened abortion, gestational hypertension, placenta abruption, severe anaemia, uterine hyperkinesia, intrauterine growth restriction) and celiac disease. According with Ludvigsson and Khashan who studied the relation with undiagnosed CD and low birth weight [29,19], a shorter gestation has on average been observed in celiac women compared to healthy controls together with a lower birthweight of celiac women babies compared to that of healthy women babies.

These results can in part be explained by: i) the high number of women who are diagnosed with celiac disease after their first pregnancy (undiagnosed CD - 85%) and ii) the low number of pregnancies (23.8%) on a gluten free diet [28-30]. Breastfeeding duration has been observed to be shorter in celiac women compared to healthy women.

The major limit of this study consists in the selection of women participating in a meeting on celiac disease who could be different, for example because they are socially more talented, from the majority of women with celiac disease who did not attend. Moreover, healthy controls could be less likely to remember specific conditions from the past than "well motivated" patients who attended a meeting targeted at their disease, like suggested by the complete absence of hypertension of pregnancy and foetal growth restriction from the control population. Most likely, also the different way by which data from controls (over the phone) were obtained could reduce the time to recall specific conditions.

In our survey we did not consider smoking status. In most studies, smoking is negatively associated with CD although Ludvigsson *et al. *in their study [31] found no association between CD and smoking in pregnant women. This could influenced risk estimates.

Finally, only 41.9% of the women with CD referred to have undergone small intestinal biopsy when they were asked for the diagnosis. This could be another weakness of the study design.

The occurrence of a significant correlation between celiac disease and menstrual cycle disorders and/or pregnancy and puerperal disorders highlighted by the present study suggests the possibility of considering celiac disease as one of the potential causes of fertility problems. Therefore celiac disease diagnostic procedures (serological screening [27,32-36]) could be routinely performed in women with reproductive disorders as a potential useful strategy to treat the same disorders and to allow for an early diagnosis of those all too often unrecognized cases of celiac disease.

## Competing interests

The authors declare that they have no competing interests.

## Authors' contributions

MD participated in the design of the study, in analysis and interpretation of data and draft the paper. FF participated in conceived of the study and in the design of the study and performed the statistical analysis. ST participated in the design of the study and acquisition of data. CAG contributed to revise the manuscript. RP conceived of the study, and participated in its design and coordination and draft the manuscript. All authors have given final approval of the version to be published.

## Pre-publication history

The pre-publication history for this paper can be accessed here:

http://www.biomedcentral.com/1471-230X/10/89/prepub

## Supplementary Material

Additional file 1 **Standardized questionnaire - Main investigated items**. The file contains the main items investigated by standardized questionnaire. Click here for file
