# Supplementary material for: Reproductive life disorders in Italian celiac women. A case-control study
Source: BMC Gastroenterol. 2010 Aug 6;10:89. doi: 10.1186/1471-230X-10-89 (PMC2928757; doi:10.1186/1471-230X-10-89)
Supplement: Additional file 1 — Standardized questionnaire - Main investigated items. The file contains the main items investigated by standardized questionnaire. [file 1471-230X-10-89-S1.DOC]

Standardized questionnaire – Main investigated items:

- Age and town of residence;
- Modality of and age at diagnosis;
- Onset symptoms (intestinal, extra-intestinal);
- Following a gluten free diet at the time of the interview;
- Age at menarche;
- History of menstrual cycle disorders (amenorrea - absence of a [menstrual period](http://en.wikipedia.org/wiki/Menstrual_period) in a woman of reproductive age; oligomenorrhea - infrequent menstrual periods, longer than 35 days; hypomenorrhea - diminution of amount or duration of menstruation; polymenorrhea - menstrual periods shorter than 21 days; dysmenorrhea - cramps or painful menstruation; metrorrhagia - vaginal bleeding not synchronized with their menstrual period; menometrorrhagia – excessive, irregularly and more frequently than normal uterine bleeding; pre-menstrual sindrome - one or more of a group of symptoms including abdominal bloating, breast tenderness, anxiety, crying spells, depression, fatigue, lack of energy, anger and irritability, changes in appetite, and varying degrees of edema that occur regularly before menstrual cycle);
- Time of onset of menstrual cycle disorders over time of onset of the pathognomonic symptoms/signs of celiac disease;
- Time of onset of menstrual cycle disorders over time of celiac disease diagnosis;
- Following a gluten free diet at the onset of menstrual cycle disorders;
- Current menstrual cycle disorders;
- Number of pregnancies and miscarriages;
- History of pregnancy problems (threats of miscarriage – vaginal bleeding associated with pain occurred during the first half of pregnancy with fetus viable (heartbeat detected and regular); gestational hypertension – systolic blood pressure ≥140 mmHg and/or a diastolic blood pressure ≥90 mmHg, in the absence of proteinuria, in a previously normotensive pregnant woman at or after 20 weeks of gestation; placenta abruption – abnormal separation of placenta from uterus after 20 weeks of gestation and prior to birth; uterine hyperkinesias – spontaneous prolonged hypertonic uterine concraction, severe anaemia – Hb < 8 g/dl; intrauterine growth restriction – estimated weight below the 10th percentile for its gestational age, *per* each full term pregnancy);
- Onset of celiac disease pathognomonic symptoms/signs. Before, after the first pregnancy;
- Celiac disease diagnosis. Before, after the first pregnancy.
- Following a gluten free diet during each pregnancy;
- Week of gestation when delivery occurred for each full term pregnancy;
- Newborn birth weight for each full term pregnancy;
- Duration of breastfeeding for each full term pregnancy.
